# Exercise training augments Sirt1-signaling and attenuates cardiac inflammation in D-galactose induced-aging rats

**DOI:** 10.18632/aging.101714

**Published:** 2018-12-23

**Authors:** Wei-Kung Chen, Ying-Lan Tsai, Marthandam Asokan Shibu, Chia-Yao Shen, Shu Nu Chang-Lee, Ray-Jade Chen, Chun-Hsu Yao, Bo Ban, Wei-Wen Kuo, Chih-Yang Huang

**Affiliations:** 1 Department of Emergency Medicine, China Medical University Hospital, Taichung, Taiwan; 2 Athletic Training and Health Department, National Taiwan Sport University, Taoyuan, Taiwan, Laboratory of Exercise Biochemistry, University of Taipei, Taipei, Taiwan; 3 Medical Research Center for Exosome and Mitochondria Related Diseases, China Medical University and Hospital, Taichung, Taiwan; 4 Department of Nursing, Meiho University, Pingtung, Taiwan; 5 Department of Healthcare Administration, Asia University, Taichung, Taiwan; 6 Department of Surgery, School of Medicine, College of Medicine, Taipei Medical University, Taipei, Taiwan; 7 Department of Biomedical Imaging and Radiological Science, China Medical University, Taichung, Taiwan; 8 Department of Endocrinology, Affiliated Hospital of Jining Medical University, Jining Medical University, Jining, Shandong, China; 9 Department of Biological Science and Technology, China Medical University, Taichung, Taiwan; 10 Graduate Institute of Chinese Medical Science, China Medical University, Taichung, Taiwan; 11 Department of Health and Nutrition Biotechnology, Asia University, Taichung, Taiwan; 12 Graduate Institute of Basic Medical Science, China Medical University, Taichung, Taiwan; * Equal contribution

**Keywords:** senescence, longevity, inflammation, swimming

## Abstract

Exercise is known to be beneficial in controlling aging associated disorders however, the consequence of long-term exercise on cardiac health among aging population is not much clear. In this study the protective effect of exercise on aging associated cardiac disorders was determined using a D-galactose-induced aging model. Eight weeks old Sprague Dawley rats were given intraperitoneal injection of 150 mL/kg D-galactose. Swimming exercise was provided in warm water for 60 min/day for five days per week. Hematoxylin and eosin staining of cardiac tissue sections revealed cardiomyocyte disarrangements in the aging rat hearts but long-term exercise training showed improvements in the cardiac histology. Exercise training also enhanced the expression levels of proteins such as SIRT1, PGC-1α and AMPKα1 that are associated with energy homeostasis and further suppressed aging associated inflammatory cytokines. Our results show that long-term exercise training potentially enhances SIRT1 associated anti-aging signaling and provide cardio-protection against aging.

## Introduction

Aging is a deteriorative process with decline in physical and functional capabilities. Aging increases the risk of diabetes mellitus (DM), cardiovascular diseases (CVD) and sarcopenia, particularly in the people with conditions such as long-term inflammation and weak responds to oxidative stress. As seen in skeletal muscles the process of aging is associated with weakening of biochemical pathways of oxidative phosphorylation and respiration in mitochondria [1-3]. During the process of aging a progressive deterioration in mitochondrial function results in the accumulation of oxidative stress causing senescence and modulations in the levels of longevity associated proteins such as SIRT1 [4,5].

SIRT1 is a redox-sensitive enzyme that regulates various cellular events including apoptosis, cell survival, endocrine signaling, chromatin remodeling, and gene transcription [6]. Activation of SIRT1 enhances mitochondrial biogenesis via its downstream protein peroxisome proliferator-activated receptor gamma coactivator 1-alpha (PGC-1α) thereby replenishing the metabolic signaling pathways and suppressing inflammatory signaling [7,8]. PGC-1α is a strong transcriptional co-activator of several transcriptional factors and nuclear receptors and it specifically controls metabolic pathways particularly, the oxidative metabolism. One of the major roles of PGC-1α is in the biogenesis of mitochondria and associated oxidative phosphorylation.

Mitochondrial biogenesis and oxidative phosphorylation in muscle, heart and fat tissue can be increased by enhancing the expression of PGC-1α [9-12]. It is also notable that while generally exercise training prevents aging-induced reduction of mitochondrial proteins and associated cellular apoptosis by providing antioxidant benefits, PGC-1α knock-out mice do not display antioxidant effects following exercise [13-15]. Similarly, acute exercise training is known to trigger the activation of the dimeric transcription factor-nuclear factor-kappaB (NFκB). NFκB is activated by various stimulants, including TNF-α. Activation of TNF-α receptor triggers IκB kinase (IKK) to phosphorylate IκB and thereby results in the release of P50/P65 subunits of NFκB complex to enable NFκB nuclear translocation [16,17]. While most studies elucidate these events in skeletal muscles, the long-term implications of exercise training in the longevity mechanisms of aging heart are not clear yet.

Long-term D-galactose IP injection is used to generate animal models for aging studies and they reproduce aging associated oxidative stress [18-21]. In this study, the cardiac effects of long-term exercise on D-galactose induced aging in Sprague-Dawley (SD) rats have been explored.

## RESULTS

### Exercise training protected cardiac characteristics

Exercise training in control rats and aging rats resulted in a notable reduction in the bodyweight however, the differences weren’t statistically significant. Meanwhile, aging or exercise training did not show any considerable change in cardiac features such as whole heart weight (WHW) and Left ventricle weight (LVW), but the WHW and LVW normalized with body weight showed significant differences (Table 1). The exercise training groups in general showed an increase in WHW/Body weight (BW) and LVW/BW when compared to their respective groups without exercise. Further interpretation of the results shows that exercise training causes a reduction in the body weight of normal as well as induced aging group rats without causing any considerable effect in the heart.

**Table 1:** Cardiac characteristics of the experimental groups.

	**Control (n=9)**	**Exercise (n=9)**	**Aging (n=9)**	**Aging + exercise (n=9)**
Body weight (BW, g)	527.33±66.41	485.80±66.41	548.67±49.11	484.17±37.32^#^
Tibia (mm)	46.13±0.34	46.62±0.77	47.57±0.58	46.78±0.17
Whole Heart weight (WHW, g)	1.49±0.14	1.63±0.32	1.59±0.09	1.64±0.17
Left ventricle weight (LVW, g)	1.03±0.10	1.11±0.22	1.13±0.06	1.13±0.11
WHW/ BW (g)	2.89±0.21	3.36±0.18^*^	3.03±0.10	3.36±0.33^#^
LVW/BW (g)	2±0.16	2.29±0.10^*^	2.16±0.10	2.31±0.18^#^
HW/Tibia (mg/mm)	32.21±2.85	34.91±6.22	33.50±2.24	35±3.10
HW/Tibia (mg/mm)	22.27±2.07	23.84±4.26	23.92±1.56	24.11±2.11

Values are means ± SD among SD rats. BW, body weight; HW, whole heart weight; LVW, left ventricular weight. *P<0.05 indicates significant differences with respect to control group. # P<0.05 indicates significant differences between aging.

### Effect of exercise on the histological changes in aging hearts

**Figure 1:**
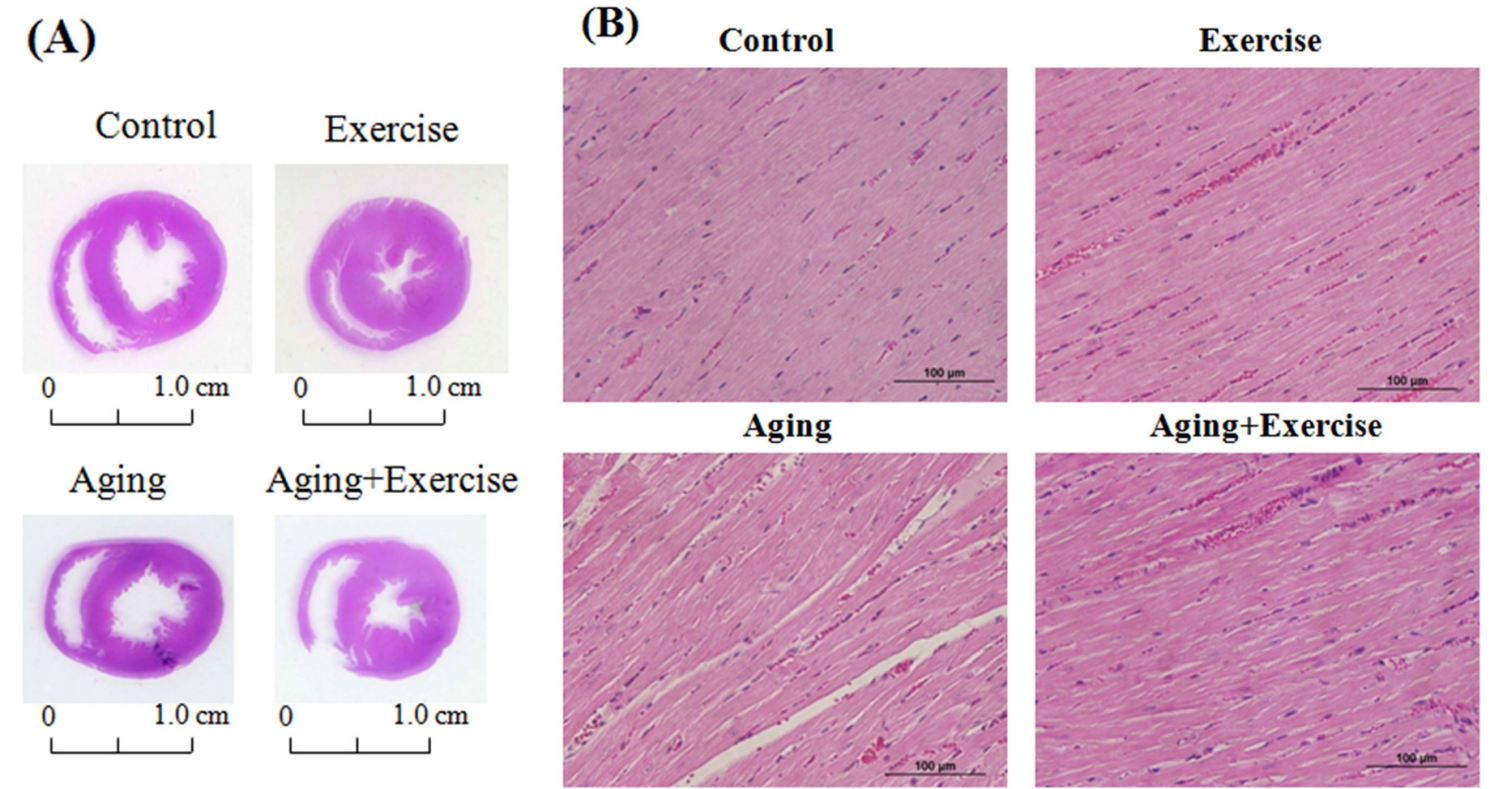
**Hematoxylin and eosin stain (H&E stain) showing the cardiac tissue architecture.** Representative histopathological analysis of cardiac sections of the left ventricles stained with hematoxylin and eosin. The hematoxylin colors basophilic structures blue-purple and the eosin colors eosinophilic structures in bright pink. The images of myocardial architecture were magnified at 400X.

After eight-weeks of swimming exercise the hearts were excised and the effects of exercise training on the histological changes were analyzed by H & E staining. Heart tissue sections from the aged rats groups (A) showed thinning of the heart muscle but the exercise trained rats (E and AE) showed thicker ventricular walls, showing phenomenon of physiological hypertrophy, when compared to the control (C) and aging (A) groups. Further analysis showed that D-galactose induced aging caused a sever disarrangement in cardiac architecture. The cardiomyocytes in aging group were disordered and showed large interstitial spaces (Figure 1). In contrary, the cardiomyocyte were orderly arranged in groups that underwent swimming exercise (E and AE).

### Exercise training suppressed mediators of inflammation in aging hearts

**Figure 2:**
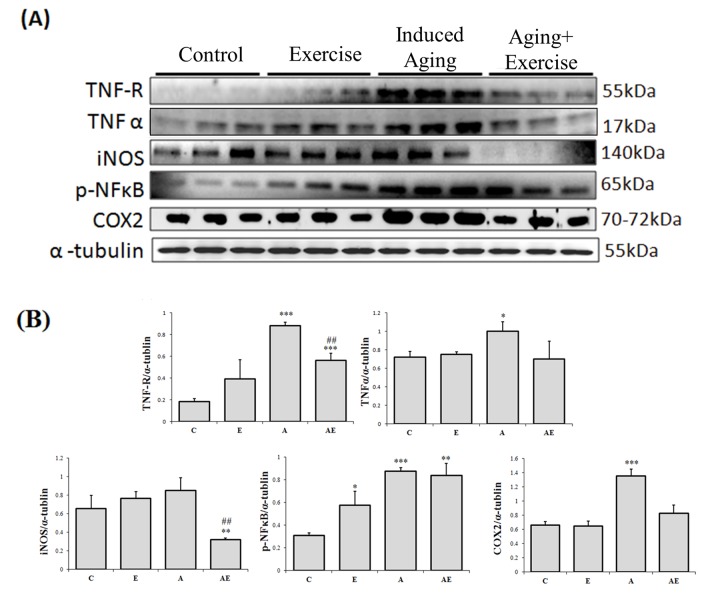
**Effect of exercise training in TNF alpha associated Inflammatory pathway.** (**A**) Representative Western blots showing protein products of TNF-R, TNF-α, iNOS, p-NFκB and COX-2 extracted from left ventricles of excised hearts (**B**) The α-tubulin was used as an internal control. (**C**) Densitometry bars showing the relative protein levels represented by mean values ± SEM. *P< 0.05, **P< 0.01, ***P< 0.001signiﬁcant differences with respect to control group. #P<0.05, ##P<0.01, ###P< 0.001 signiﬁcant differences with respect to aging group.

The western blotting assay was used to assess the effect of swimming exercise on inflammation associated proteins and the results show that cardiac TNF-R, TNF-α, iNOS and COX-2 proteins expressions did not show any significant difference in the control rats (C) and the exercise training (E) rats (Figure 2). As expected, D-galactose induced aging rats (A) showed a significant increase in the levels of TNF-R, TNF-α, pNFκB and COX-2 levels when compared to that of the control (C). Interestingly exercise training significantly reduced the levels of the inflammatory markers in the aging rats. Notably, the COX-2 expression in rat hearts was 2.25 folds in D-galactose induced-aging rats compared with control, and those aging rats subjected to swimming exercise showed only 1.33 folds to that of the control.

### Exercise training enhances longevity

**Figure 3:**
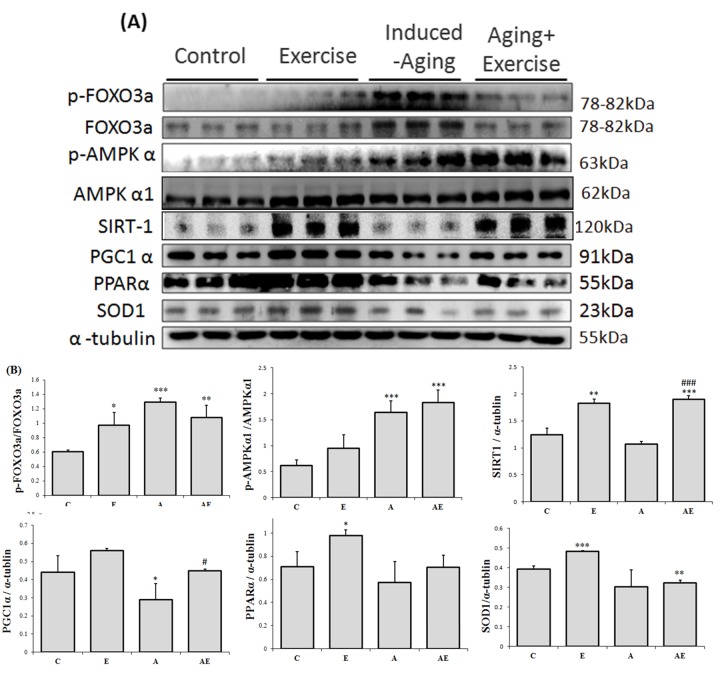
**Effect of exercise training on APK associated cellular homeostasis.** (**A**) Representative Western blots showing modulation in the levels of p-FOXO1, FOXO3a, p-FOXO3a, p-AMPKα1, AMPKα1, SIRT1, PGC-1α, PPARα, and SOD-1 extracted from the left ventricles of excised hearts. The α-tubulin was used as an internal control. (**B**) Densitometric analysis showing relative changes in protein levels represented by mean values ± SEM. *P< 0.05, **P< 0.01, ***P< 0.001signiﬁcant differences with respect to control group. #P<0.05, ##P<0.01, ###P< 0.001 signiﬁcant differences with respect to aging group.

Analysis on the levels of AMPK and its downstream cellular longevity associated proteins revealed that exercise training in normal rats elevated the levels of pFOXO3a, of pFOXO3a, levels (figure 3). Meanwhile aging rats showed further elevation of pFOXO3a and pAMPKα1 levels and reduced levels of SIRT1, PGC-1α, PPARα and SOD1 however exercise training caused reversion in the levels of pFOXO3a, SIRT1 and PGC-1α and there was no notable difference in the levels of pAMPKα1, PPARα and SOD1 when compared to the aging group.

## DISCUSSION

Previous studies show that various conditions such as hypertension, obesity and diabetes cause cardiac abnormalities in rats. Inflammatory response associated with these conditions trigger cardiac hypertrophy and apoptosis that subsequently result in cardiac dysfunction. Several alternative strategies have been shown to provide cardio-protection against various pathological conditions [22-29]. Exercise training is also known to provide cardio-protection in conditions such as ischemia-reperfusion injury [30,31].

**Figure 4:**
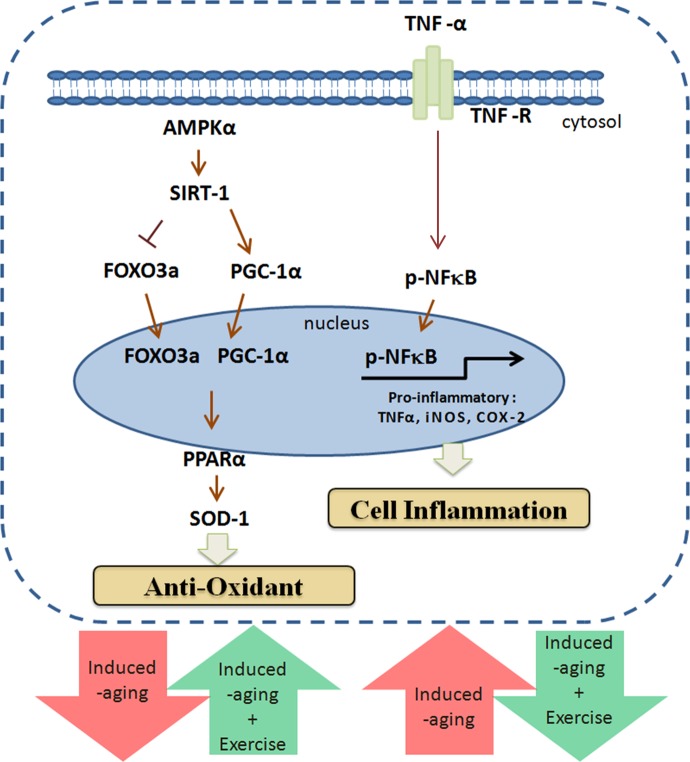
**Exercise enhances metabolic adaptation and attenuates inflammation in aging hearts.** Long-term exercise training enhances SIRT1, PGC-1α and AMPK in aging hearts to provide protection against aging associated damages.

The biochemical and physiological changes in the D-galactose induced aging rat include increase in senescence, oxidative stress, mitochondrial dysfunction and cardiac apoptosis therefore the model very much represents the natural aging animal models [32]. Our results also show that aging group undergoes a 6% increase in whole heart weight and 9% increase in left-ventricle (LV) weight. Increasing whole heart and left-ventricle weight are common phenomenon associated with hypertrophy in conditions such as high blood pressure, obesity and aging [33-35]. Thus, the effects of D-galactose induced aging in heart could be correlated as a potential risk factor of cardiomyopathy. Exercise training is also known to improve heart function from cardiomyopathy conditions as reported by Boyne et al., 2013 [36]. Further, the reduction of the LV weight ratio observed in myocardial infarction animal models was found to be improved with respect to cardiac function and deterioration in cardiac remodeling. The effects of exercise were also correlated with improvements in the inflammatory profile in the chronic heart failure animal models [37]. Exercise training has been shown to provide anti-inflammatory effects as inferred from upregulated NFκB expression and endothelial vascular relaxation [38]. Our results show that expression levels of inflammatory markers such as TNF-α, NFκB, COX-2 and iNOS were steadily increased in aging group but were effectively reverted in exercise training groups therefore it can be interpreted that exercise training could potentially regulate and maintain the cellular homeostasis with respect to inflammation and oxidative stress (Figure 4) [39]. Interestingly, the compensative expression of p-NFκB was observed in D-galactose induced aging when combined with long-term exercise training. The possible reasons might be due to the variation in time and frequency of the exercise training [40].

SIRT1, a deacetylase is involved in the metabolic adaptation of muscle tissue to endurance exercise possibly due to the imbalances in the cytosolic NAD+/NADH ratio caused by muscle contraction [41]. Increase in SIRT1 levels facilitate such metabolic adaptation of muscles [42] and also regulate age-associated changes through various mechanisms that include enhancing the mitochondria biogenesis by deacetylation and activation of PGC-1α, containing oxidative stress and promoting survival signals by activation of FOXO family proteins, reducing apoptosis and proliferation and alleviating pro-inflammatory response of aging [43-45]. Exercise also intensifies the contraction of cardiac muscles which may facilitate SIRT1 upregulation and our results also correlates exercise training with elevated SIRT1 levels.

Aging is associated with increasing stressful conditions like elevated ROS levels and inflammation which effectively trigger cell death which may result in the disarrangement in the cardiomyocyte arrangement causing reduction in the efficiency of the heart muscles [46]. The histological assessment performed in this study shows that exercise training improved the cardiomyocyte arrangement reflecting an improvement in the cardiac function.

In oxidative stress conditions FOXO3a that exist in nucleus induces the expression of inflammatory proteins. The deacetylase SIRT1 removes the acetyl group on the FOXO3a and attenuates its function, thereby protects the cell and also stabilizes the nuclear DNA [47]. The elevated levels of FOXO3a in D-galactose induced aging rat observed in this study were reduced with exercise training which was correlated with elevated SIRT1 levels which potentially contributes to deacetylase activity in cardiac cells.

The energy consumption for exercise is usually subject to glucose transport through AMPK independent pathway. In our results, the AMPK expression was higher in normal rats with exercise training but, interestingly, the p-AMPK level was higher in aging rats and was further increased in the aging rats with exercise training. The results suggest an energy demand that existed in D-galactose induced aging rat hearts, and the AMPK independent pathway was not regulated after exercise training. AMPK is an energy sensor and since it also increases the intracellular NAD+ levels its activity is correlated with SIRT1-enhancment [48]. However since SIRT1 can also activate the predominant AMPK kinase LKB-1, it is also identified as an upstream activator of AMPK. Therefore in certain conditions the effects of exercise are mediated through LKB1/AKT activation and in some they are mediated through SIRT1 induced deacetylation. In this study, although a correlation exists in the levels of AMPK and SIRT1, the active pAMPK levels in the aging groups seem to be independent of SIRT1 levels. Therefore our results also show that, although when AMPK and SIRT1 levels are generally inter-dependable [49] they are independent in some conditions such as aging.

In conclusion, our results demonstrate that long-term swimming exercise can enhance SIRT1, PGC-1α and AMPK in cardiac cells of aging rats and thereby facilitate metabolic adaptation in the heart and attenuates the activation of pro-inflammatory mediators and collectively ameliorates aging induced hypertrophic effects.

## MATERIALS AND METHODS

### Animal models

Three months old male Sprague Dawley (SD) rats were purchased from BioLASCO (Taipei, Taiwan). The rats were housed in cages with temperature maintained at 24 ± 2 °C, 55 ± 10% of humidity and 12 h light-dark cycle and were provided a standard laboratory diet (Lab Diet 5001; PMI Nutrition International Inc., Brentwood, MO, USA) and drinking water ad libitum. After one week adaptation the rats were divided into four groups: Control group (C, n=9), Exercise training group (E, n=9), Aging group (A, n=9), Aging group with Exercise training (AE, n=9). The control rats were given 0.9% saline (IP, 1 mL) and the aging induced groups (A and AE) received D-galactose (150 mg/kg body weight) for eight weeks. The swimming training protocol was performed as mentioned in other reports [50]. The Initial exercise training during the first two weeks was given for 20 min/day for five times a week. In the third week the timing of the swimming exercise training time was increased to 30 min per day and from fourth to eighth week the swimming exercise training was given for 60 min/day. The rats swam individually in a water bath (60 × 90 cm, 50 cm depth) with the water temperature maintained at 35 ± 1°C. All protocols were approved by the Institutional Animal Care and Use Committee of China Medical University, Taichung, Taiwan. The study followed the principles of laboratory animal care (NIH publication).

### Protein extraction and western blotting

Heart tissue extracts obtained by homogenizing in a lysis buffer (0.05M of pH 7.4 Tris-HCl, 1 mM EDTA, 0.15 M NaCl, 1% NP-40, 0.25% deoxycholic acid) at a ratio of 100 mg tissue/1 mL buffer. The homogenates were kept on ice and the proteins in the supernatants were collected by centrifugation at 13,000 rpm for 40 min and stored at -80 °C. Protein concentrations in heart tissue extracts were determined by the Lowry protein assay. Western blotting was performed following methods mentioned in other reports with slight modification [51]. The protein samples were separated in 8%-12% SDS polyacrylamide gel electrophoresis (SDS-PAGE) under 75 V for 120 min. The separated proteins were subsequently transferred to PVDF membranes (GE healthcare limited, Buckinghamshire, UK) under 50 V for 3 h. The membranes were blocked in 3% bovine serum albumin (BSA) in tris-buffered saline and were then hybridized with primary antibodies including TNF-R (SC-1070, Santa Cruz Biotechnology, California, California, USA), TNF-α (SC-1350, Santa Cruz Biotechnology, California, California, USA), iNOS(610328, BD, New Jersey, USA), p-NFκB (#3033L, Cell Signaling, Maryland, USA) , COX-2(SC-1745, Santa Cruz Biotechnology, California, USA),α-tubulin (SC-5286, Santa Cruz Biotechnology, California, USA), p-FOXO1(#9464, Cell Signaling, Maryland, USA), FOXO3a(#2497, Cell Signaling, Maryland, USA), p-FOXO3a(#9466, Cell Signaling, Maryland, USA), p-AMPKα1(#2535, Cell Signaling, Maryland, USA), AMPKα1(SC-19128, Santa Cruz Biotechnology, California, USA), SIRT-1(SC-74465, Santa Cruz Biotechnology, California, USA), PGC-1α(AF-1817a, ABGENT, San Diego , USA ), PPAR-α(SC-9000, Santa Cruz Biotechnology, California, USA), SOD1(SC-8647, Santa Cruz Biotechnology, California, USA), Finally, the blots were hybridized with horseradish peroxidase-labeled secondary antibodies and pictures were then taken with Fujifilm LAS-3000 (GE healthcare UK limited., Buckinghamshire, UK).

### Hemotoxyline and eosin staining

The embedded rat heart sections were cut into 0.2 µm thick slices and were deparaffinized by immersing in xylene and rehydrated by subjecting to graded alcohol (100%-60%). All slices were stained with hematoxylin and eosin (H&E) and photomicrographs were obtained using Zeiss Axiophot microscopes.

### Statistical analysis

The results shown are means ± SD of three independent experiments. Statistical differences were evaluated by ANOVA one-way analysis of variants. p< 0.05 was considered statistically significant
